# Molecular remodeling of adipose tissue is associated with metabolic recovery after weight loss surgery

**DOI:** 10.1186/s12967-022-03485-6

**Published:** 2022-06-23

**Authors:** Annie Bouchard-Mercier, Juan de Toro-Martín, Mélanie Nadeau, Odette Lescelleur, Stéfane Lebel, Denis Richard, Laurent Biertho, André Tchernof, Marie-Claude Vohl

**Affiliations:** 1 School of Nutrition and Centre Nutrition, Santé et Société (NUTRISS)-Institut sur la nutrition et les aliments fonctionnels (INAF), Université Laval, Pavillon des Services (suite 2729K), 2440 Hochelaga Blvd, Quebec City, QC G1V 0A6 Canada; 2 Centre de recherche de l’institut universitaire de cardiologie et de pneumologie de Québec (IUCPQ), 2725 chemin Sainte-Foy, Quebec City, QC G1V 4G5 Canada

**Keywords:** Whole genome, Transcriptomic, Methylomic, Obesity, Bariatric surgery, Remission, Type 2 diabetes, Dyslipidemia, Subcutaneous adipose tissue

## Abstract

**Background:**

Bariatric surgery is an effective therapy for individuals with severe obesity to achieve sustainable weight loss and to reduce comorbidities. Examining the molecular signature of subcutaneous adipose tissue (SAT) following different types of bariatric surgery may help in gaining further insight into their distinct metabolic impact.

**Results:**

Subjects undergoing biliopancreatic diversion with duodenal switch (BPD-DS) showed a significantly higher percentage of total weight loss than those undergoing gastric bypass or sleeve gastrectomy (RYGB + SG) (41.7 ± 4.6 vs 28.2 ± 6.8%; p = 0.00005). Individuals losing more weight were also significantly more prone to achieve both type 2 diabetes and dyslipidemia remission (OR = 0.75; 95%CI = 0.51–0.91; p = 0.03). Whole transcriptome and methylome profiling showed that bariatric surgery induced a profound molecular remodeling of SAT at 12 months postoperative, mainly through gene down-regulation and hypermethylation. The extent of changes observed was greater following BPD-DS, with 61.1% and 49.8% of up- and down-regulated genes, as well as 85.7% and 70.4% of hyper- and hypomethylated genes being exclusive to this procedure, and mostly associated with a marked decrease of immune and inflammatory responses. Weight loss was strongly associated with genes being simultaneously differentially expressed and methylated in BPD-DS, with the strongest association being observed for *GPD1L* (r^2^ = 0.83; p = 1.4 × 10^–6^).

**Conclusions:**

Present findings point to the greater SAT molecular remodeling following BPD-DS as potentially linked with higher metabolic remission rates. These results will contribute to a better understanding of the metabolic pathways involved in the response to bariatric surgery and will eventually lead to the development of gene targets for the treatment of obesity.

*Trial registration* ClinicalTrials.gov NCT02390973.

**Supplementary Information:**

The online version contains supplementary material available at 10.1186/s12967-022-03485-6.

## Introduction

According to the World Health Organization, obesity prevalence has almost tripled since 1975, with over 650 million adults worldwide suffering from this condition in 2016 [1]. Obesity is related to many comorbid conditions including cardiovascular disease, type 2 diabetes, non-alcoholic fatty liver disease, obstructive sleep apnea, reproductive dysfunction, musculoskeletal disorders, certain types of cancer, as well as psychosocial consequences [2, 3]. Unfortunately, non-surgical treatment of severe obesity using modalities such as diet, exercise, or even pharmacological treatment have low to moderate success rates, especially when considering medium- to long-term weight maintenance [4]. Bariatric surgery, also known as metabolic surgery, emerged as an effective therapy for individuals with severe obesity to achieve significant sustainable weight loss, as well as to reduce the associated comorbidities [3, 5, 6]. The term metabolic surgery was proposed to acknowledge the physiological changes induced by these approaches, which contribute to a more favorable metabolic profile following the surgery, beyond the traditional view that these surgeries induce beneficial effects through weight loss alone [6–8]. Yet, these physiological changes are still not fully elucidated.

Among the different types of bariatric surgery, sleeve gastrectomy (SG) is one the most common and simple from a surgical standpoint, consisting of a surgical removal of about 80 percent of the stomach along the greater curvature, physically restricting food intake. In addition to reducing gastric volume, Roux-en-Y gastric bypass (RYGB) also decreases the efficiency of nutrient absorption in the small intestine by creating a little gastric pouch directly connected to the jejunum, bypassing the duodenum. Biliopancreatic diversion with duodenal switch (BPD-DS) is the most complex of these bariatric procedures, combining gastric restriction induced by SG and a more marked malabsorption than that observed after RYGB. Briefly, a pylorus-preserving SG procedure is combined with a transection of the duodenum near the pylorus creating an alimentary limb that connects the biliary limb near the ileo-cecal valve to create a short common channel where nutrients are absorbed [9].

Epigenetics may help in our understanding of how an individual will respond to bariatric surgery as the latter may be viewed as an environmental factor modifying the epigenome, although certain epigenetic marks may be inheritable [10, 11]. DNA methylation is the most widely investigated epigenetic mechanism, and some studies have predicted weight loss or weight regain after bariatric surgery according to baseline gene methylation [12, 13]. Bariatric surgery also promotes modifications in methylation profiles of individuals with obesity, which are more akin to those who are lean [14, 15]*.* Some authors have also observed lower overall methylation levels after RYGB in subcutaneous adipose tissue (SAT) [16, 17]. Conversely, more recent studies have observed higher methylation levels at cytosine-phosphate-guanine dinucleotides, or CpG sites, after RYGB and SG procedures in peripheral blood [15], as well as higher global methylation levels in skeletal muscle after RYGB [18]. These discrepancies may partly be explained by tissue-specific DNA methylation [19]. As compared to RYGB and SG, BPD-DS is a surgery that creates a greater nutrient malabsorption, due to a reduced length of the intestinal segment allowed for absorption, especially impacting dietary lipids [20, 21], and it has been proven to be particularly effective among individuals with severe obesity [22, 23]. By contrast, the impact of BPD-DS on the epigenetic profile of SAT is almost completely unknown.

The SAT is much more than a site of storage for excess energy intake, and it is recognized to play a crucial role in energy homeostasis control and inflammation [24]*.* Although significant modifications occur in the SAT after surgically induced weight loss [25, 26], only few studies have investigated gene expression changes in this tissue [27–31]. Genes previously identified as differentially expressed in SAT following bariatric surgery are involved in lipid and energy metabolism, inflammatory and immunological responses, insulin signaling, cell differentiation, oxidative stress regulation and gene transcription [27, 30]. A recent study [32] has also observed long-term effects of RYGB on gene expression in abdominal SAT, with enriched pathways related to lipid metabolism, fat cell differentiation and immune response. Again, most of the studies examining gene expression changes in SAT have been conducted among individuals with obesity who had undergone RYGB or SG, but none had investigated the impact of BPD-DS on the transcriptomic SAT profile [27–31].

Examining molecular changes taking place following a weight-loss procedure may then help in gaining further insight into its distinct metabolic impact and may eventually aid in targeting the most beneficial intervention. Thus, our objective was to compare methylation and gene expression changes in SAT following three different types of bariatric surgeries, namely BPD-DS, RYGB and SG. We hypothesized that the more metabolically effective BPD-DS leads to greater modifications at the methylation and gene expression level than the extensively studied RYGB and SG bariatric surgeries.

## Results

### *BPD-DS induced a more pronounced weight loss than RYGB* + *SG*

**Fig. 1:**
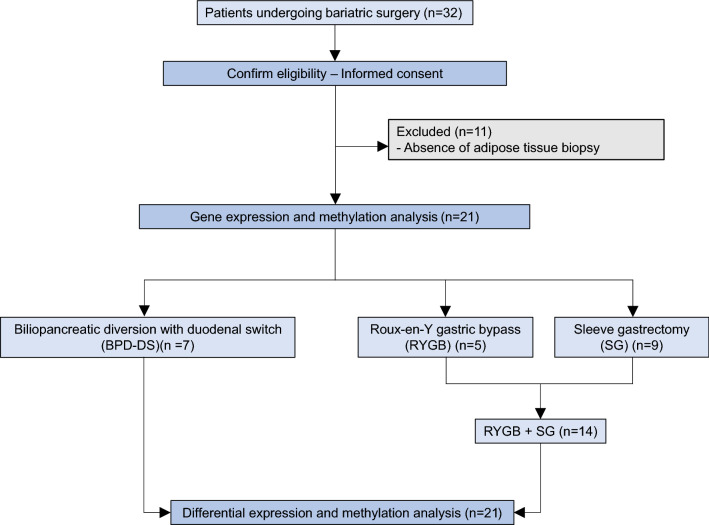
Flow diagram of study participants

Of the 21 participants, 7 underwent BPD-DS, 5 RYGB and 9 SG (Fig. 1). Altogether, participants from the entire cohort showed a preoperative mean body mass index (BMI) of 44.4 kg/m^2^ ± 6.1, with a %TWL of 32.7% ± 8.9 at 12 months following the surgery. Due to the similar mean percent total weight loss (%TWL) shown by both RYGB (31.9% ± 6.4) and SG (26.2% ± 6.5) participants, these two groups were combined into the RYGB + SG group and compared to participants undergoing BPD-DS. Table 1 shows detailed information about the anthropometric measurements of BPD-DS and RYGB + SG surgery groups. Characteristics of subjects from the three different surgery procedures, BPD-DS, RYGB and SG, are detailed in Additional file 1: Table S1. When comparing BPD-DS and RYGB + SG surgery groups, we found that BPD-DS participants had significantly higher mean body weight, BMI, waist circumference and fat mass than RYGB + SG participants at baseline (Table 1). The proportion of men and women was not significantly different between surgery groups (p = 0.4) Following the surgery, at 12 months postoperative, BPD-DS participants had significantly higher mean delta BMI and %TWL than RYGB + SG subjects. No significant differences were found according to mean body weight, BMI, percent excess weight loss (%EWL), waist circumference or body composition (fat and lean mass). BPD-DS participants showed significantly lower neck circumference at 12 months postoperative, as compared to RYGB + SG participants (Table 1). No significant differences were found for adipocyte size 12 months following the surgery between BPD-DS and RYGB + SG groups, but participants undergoing SG showed significantly larger adipocytes than those who had BPD-DS and RYGB separately (Additional file 1: Table S1; Figure S1).

**Table 1:** Characteristics of participants

Parameters	Preoperative		P-value	Postoperative		P-value
	RYGB + SG	BPD–DS		RYGB + SG	BPD–DS	
N (male)	14 (8)	7 (2)	0.4	–	–	–
Age (years)	54.2 ± 7.8	47.8 ± 7.1	0.1	–	–	–
Height (cm)	167.8 ± 8.7	167.1 ± 8.9	0.9	–	–	–
Body weight (kg)	117.2 ± 17.3	139.8 ± 15.3	0.009	83.9 ± 15	81.4 ± 10.0	0.7
BMI (kg/m^2^)	41.5 ± 4.1	50.2 ± 5.5	0.004	29.7 ± 4.3	29.3 ± 4.1	0.8
ΔBMI	–	–	–	11.8 ± 2.9	-20.9 ± 2.9	0.00002
%TWL	–	–	–	28.2 ± 6.8	41.7 ± 4.6	0.00005
%EWL	–	–	–	65.2 ± 18.8	78.6 ± 15.1	0.1
Neck circ. (cm)	44.3 ± 2.7	45.1 ± 3.3	0.6	38.6 ± 2.9	35.2 ± 2.8	0.03
Waist circ. (cm)	134.1 ± 10.1	147.9 ± 9.8	0.01	105.2 ± 11.7	104.1 ± 8.5	0.8
Fat mass (kg)	55.3 ± 12.6	78.3 ± 7.5	0.00006	25.5 ± 11.3	26.8 ± 9.1	0.8
Fat free mass (kg)	63.9 ± 11	66.1 ± 6.2	0.6	57 ± 11.0	56.2 ± 5.1	0.8
Adipocyte size (µm)	85.5 ± 8.4	88.8 ± 5.1	0.3	64.8 ± 8.9	58.5 ± 7.9	0.1
SBP	137.4 ± 18.1	143.1 ± 16.5	0.5	133.6 ± 20.8	129.9 ± 12.3	0.6
DBP	81.8 ± 6.9	82.7 ± 6.8	0.8	78.6 ± 13.5	74.3 ± 8.8	0.4

N number of participants, BMI body mass index, ΔBMI delta BMI, %TWL percentage of total body weight loss, %EWL percentage of excess body weight loss, Circ circumference. SBP and DBP stand for systolic and diastolic blood pressure, respectively

### Total remission was significantly higher in BPD-DS

All participants included in the study had type 2 diabetes before the surgery, with a mean duration of 8.4 ± 8.3 years since diagnostic, based on the Canadian Diabetes Association guidelines [33] (fasting plasma glucose ≥ 7.0 mmol/L, or glycated hemoglobin (HbA1c) ≥ 6.5%, or 2 h glycemia in a 75 g oral glucose tolerance test (OGTT) ≥ 11.1 mmol/L, or random glycemia ≥ 11.1 mmol/L). Type 2 diabetes was treated with either oral hypoglycemic drugs (n = 16, 76%), insulin (n = 4, 19%), or diet alone (n = 1, 7%). Most of participants also presented other comorbidities such as hypertension (n = 18, 86%), dyslipidemia (n = 18, 86%), or cardiovascular disease (n = 5, 24%). At 12 months postoperative, 13 participants (62%) were in complete type 2 diabetes remission, 4 were (19%) in partial remission and 4 (19%) had improvements in their glycemic control. For dyslipidemia, one participant from BPD-DS and two from RYGB + SG had no preoperative data, leaving 12 (67%) participants with complete remission, and 6 (33%) with improved, unchanged, or abnormal values. Following BPD-DS, 100% of participants achieved complete remission of type 2 diabetes (7 out of 7) and dyslipidemia (6 out of 6), while this percentage was 50% following RYGB + SG for type 2 diabetes (7 out of 14) (Fisher’s p = 0.05) and dyslipidemia remission (6 out of 12) (Fisher’s p = 0.05). Consequently, total remission rate was significantly different between the two surgery groups, with 100% of participants showing total remission following BPD-DS (6 out of 6), as compared to only 25% (3 out of 12) following RYGB + SG (Fisher's p = 0.009).

### Extensive transcriptomic remodeling occurred in the SAT of BPD-DS participants

**Fig. 2:**
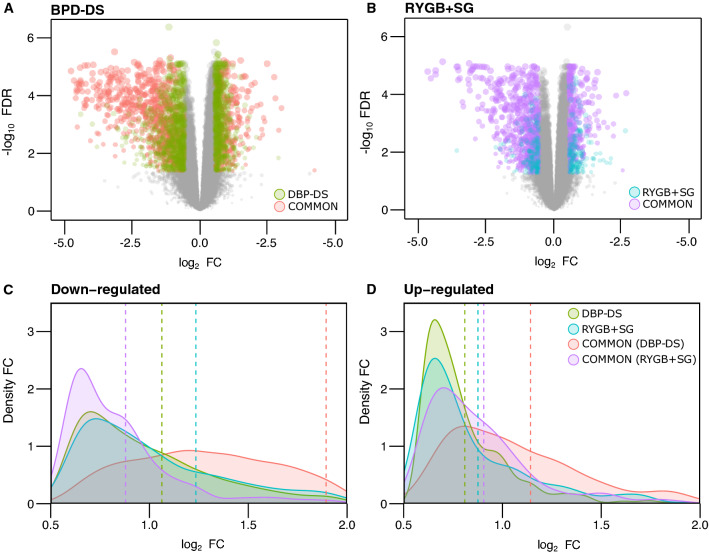
Gene expression changes in subcutaneous adipose tissue were more pronounced following BPD-DS. Panel **A** shows differentially expressed genes (DEGs) exclusive for BPD-DS (green dots) and common to both surgery groups (red dots). Panel **B** shows DEGs exclusive for RYGB-DS (blue dots) and common to both surgery groups (purple dots). DEGs were considered significant when false discovery rate (FDR)-corrected p-value < 0.05 and fold change (FC) > 1.5. Panels **C** and **D** show density plots of FC distribution among down-regulated and up-regulated DEGs, respectively. Green and blue colors stand for DEGs exclusive for BPD-DS and RYGB + SG, respectively. Red and purple colors stand for DEGs common to both surgery groups but showing the specific FC distribution for each BPD-DS and RYGB + SG surgery, respectively. Dotted lines stand for mean FC values for each surgery group

To examine the impact of bariatric surgery on SAT gene expression, we first compared SAT transcriptomic profiles between baseline and 12 months postoperative for each surgery group. First, we found that most of differentially expressed genes (DEGs) were exclusive to the BPD-DS group. Concretely, 713 up- and 943 down-regulated DEGs, representing 61.1% and 49.8% from the total DEGs, respectively (Fig. 2A). Conversely, only a few were exclusive to the RYGB + SG surgery group, with 170 (14.6%) up- and 169 (8.9%) down-regulated DEGs (Fig. 2B). Thus, there were approximately four times more up-regulated and six times more down-regulated DEGs exclusive to BPD-DS than to RYGB + SG. Regarding DEGs common to both surgery groups, there were three times more down-regulated than up-regulated genes, that is 782 (41.3%) down- versus 283 (24.3%) up-regulated DEGs (Fig. 2A–B). Interestingly, among common DEGs, the mean fold change of down-regulated DEGs was more than 50% greater in the BPD-DS group (1.89 log_2_ FC) than in the RYGB + SG group (1.26 log_2_ FC) (Fig. 2C), and approximately 35% greater for up-regulated DEGs (1.14 versus 0.85 log_2_ FC) (Fig. 2D).

### Most of the differentially methylated genes were exclusive to BPD-DS

**Fig. 3:**
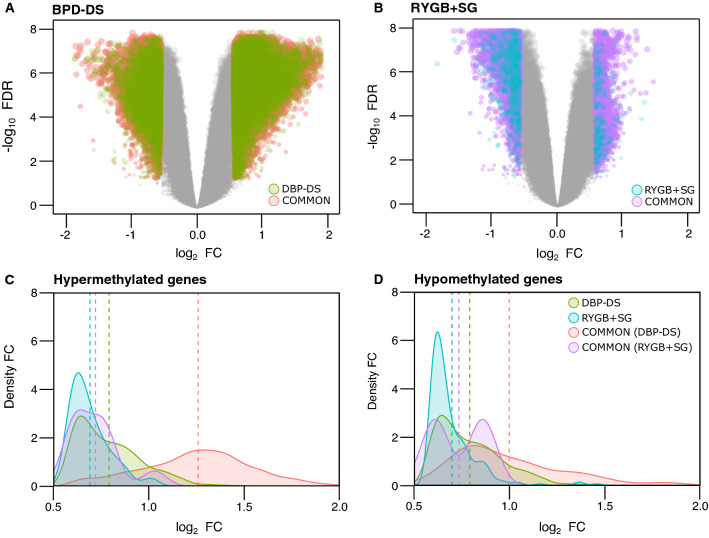
Most of gene methylation changes in subcutaneous adipose tissue occur following BPD-DS. Panel **A** shows differentially methylated genes (DMGs) exclusive for BPD-DS (green dots) and common to both surgery groups (red dots). Panel **B** shows DMGs exclusive for RYGB-DS (blue dots) and common to both surgery groups (purple dots). DMGs were defined as loci with at least one differentially methylated CpG site (false discovery rate (FDR)-corrected p-value < 0.05 and fold change (FC) > 1.5. Panels **C** and **D** show density plots of FC distribution among hypermethylated and hypomethylated DMGs, respectively. Green and blue colors stand for DEGs exclusive for BPD-DS and RYGB + SG, respectively. Red and purple colors stand for DMGs common to both surgery groups but showing the specific FC distribution for each BPD-DS and RYGB + SG surgery, respectively. Dotted lines stand for mean FC values for each surgery

Differentially methylated loci containing at least one differentially methylated CpG site were examined in SAT at baseline and 12 months following the surgery for each group. As for the DEGs, most of the differentially methylated genes (DMGs) were exclusive to BPD-DS, with 8 094 DMGs being significantly hypermethylated and 6 369 hypomethylated, representing 85.7% and 70.4% of the total DMGs (Fig. 3A). Only a few DMGs were exclusive to the RYGB + SG group, with 114 (1.2%) hypermethylated and 769 (8.5%) hypomethylated DMGs (Fig. 3B). Furthermore, the mean fold change of hypermethylated DMGs common to both surgery groups was 50% greater following BPD-DS (1.26 log_2_ FC), as compared to RYGB + SG (0.68 log_2_ FC) (Fig. 3C), as well as for hypomethylated DMGs, although to a lesser extent (Fig. 3D).

### Immune-related pathways were differentially enriched and down-regulated in the SAT of BPD-DS participants

**Fig. 4:**
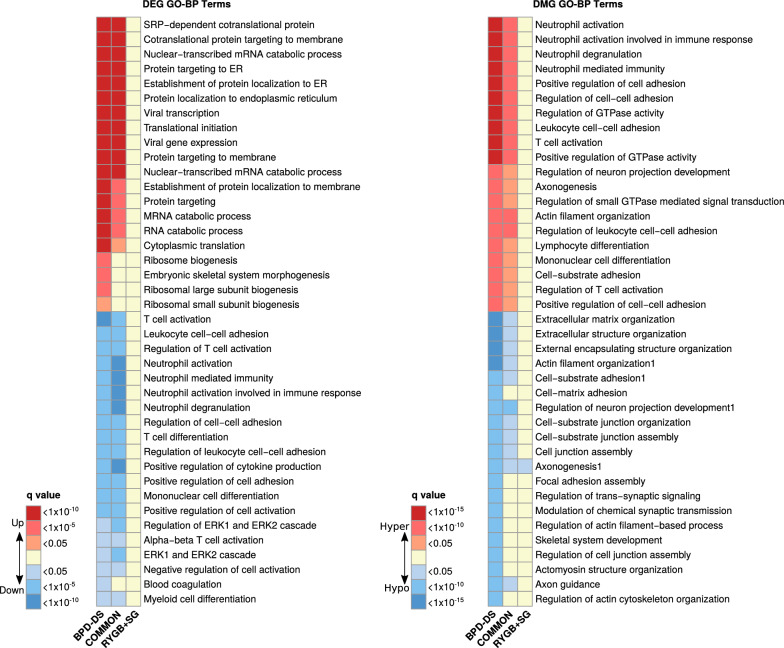
Immune-related pathways were markedly down-regulated following bariatric surgery. Left panel shows top Gene Ontology-Biological Process (GO-BP) terms significantly enriched with up-regulated (red blocks, up) and down-regulated (blue blocks, down) differentially expressed genes (DEGs). Right panel shows top GO-BP terms significantly enriched with hypermethylated (red blocks, up) and hypomethylated (blue blocks, down) differentially methylated genes (DMGs). Each column represents pathways enriched with DEGs specific to BPD-DS, RYGB + SG or common to both surgery groups. Pathways were considered significantly enriched when composed with at least 20 DEGs or DMGs and with FDR-adjusted p-value < 0.05

Pathway enrichment analysis was used to gain further insights into the biological processes significantly enriched with both DEGs and DMGs. A total of 15 and 225 pathways were found to be significantly enriched with up- and down-regulated DEGs common to both surgeries, whereas 27 and 122 were significantly enriched with up- and down-regulated DEGs exclusive to the BPD-DS group. In contrast, we did not find any pathway significantly enriched with up- or down-regulated DEGs exclusive to RYGB + SG. For illustrative purposes, most significantly enriched pathways exclusive to BPD-DS are shown in Fig. 4A and compared to pathways enriched with common and RYGB + SG exclusive DEGs. First, it is worth noting that most of up-regulated pathways exclusive to BPD-DS were also enriched with DEGs common to both surgery groups and were linked to the establishment of protein localization, RNA catabolic processes and protein translation, among others. On the other hand, down-regulated pathways were much more numerous with both BPD-DS and common DEGs, as already mentioned, and almost all the top enriched pathways were shared between these two groups (Fig. 4A). Down-regulated pathways were mostly related to immune and inflammatory biological processes, such as neutrophil-mediated immunity, response to bacterial molecules, leukocyte chemotaxis or cytokine production.

It is worth highlighting that, among the top metabolic pathways significantly enriched with hypermethylated DMGs exclusive to BPD-DS, most of them were related to immunity and inflammation, as previously shown for down-regulated DEGs (Fig. 4B). Similarly, most of these pathways were also found to be significantly enriched with hypermethylated DMGs common to both surgery groups. Again, no pathways were significantly enriched with hypermethylated DMGs exclusive to the RYGB + SG group. On the other hand, extracellular matrix and structure organization, actin filament organization or cell-substrate adhesion were among the top biological processes significantly enriched with hypomethylated DMGs exclusive to BPD-DS, with only half of them being also enriched with DMGs common to both surgeries (Fig. 4B). Only the axonogenesis pathway was significantly enriched in both BPD-DS and RYGB + SG surgery groups.

### Most of genes that were both differentially expressed and methylated were found in the SAT of BPD-DS participants

**Fig. 5:**
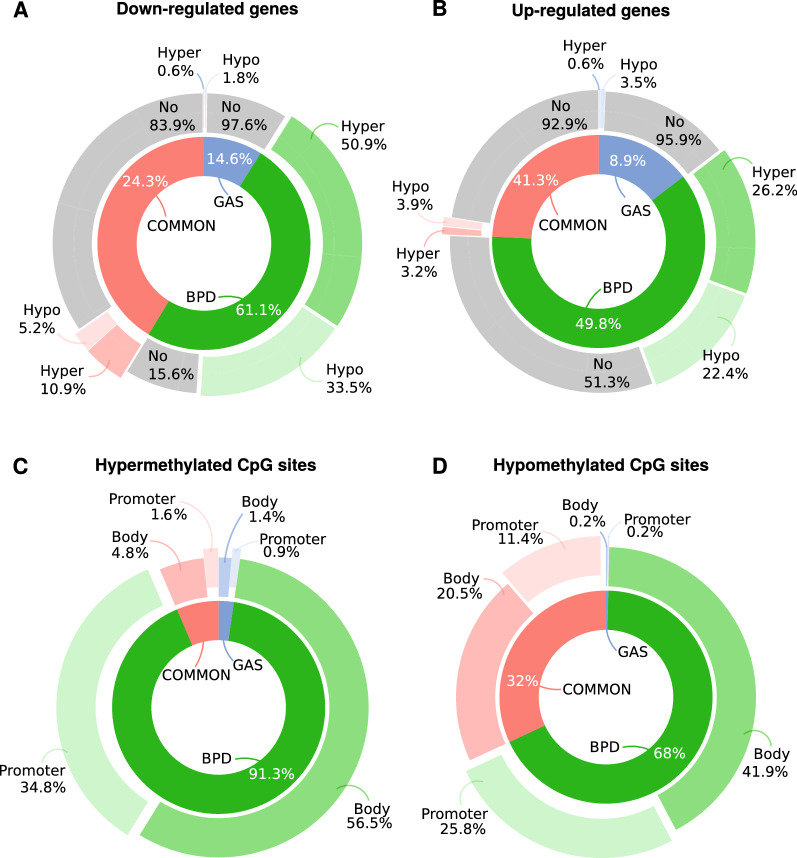
Genes being simultaneously differentially expressed and methylated largely belonged to the BPD-DS surgery group.** A** and **B** show respectively the proportion of differentially expressed genes (DEGs) down- and up-regulated that are simultaneously identified as differentially methylated genes (DMGs). The proportion of DEGs common to both surgery groups (COMMON), as well as exclusive to BPD-DS (BPD) and RYGB + SG (GAS) is shown in the inner ring. The proportion of hypermethylated and hypomethylated DMGs is shown in the outer ring. **C** and **D** show respectively the proportion of hypermethylated and hypomethylated CpG sites located within body or promoter regions of genes being simultaneously DEGs and DMGs. The proportion of genes being simultaneously DEGs and DMGs is shown in the inner ring, and the proportion of CpG sites for each gene location and surgery group is shown in the outer ring

We assessed whether gene expression changes were taking place in the same genes in which epigenetic modifications were also observed. Globally, almost 70% of the DEGs exclusive to BPD-DS surgery group were also differentially methylated. Concretely, most of down-regulated (796, 84.4%; Fig. 5A) and almost half of up-regulated DEGs (347, 48.6%; Fig. 5B) exclusive to the BPD-DS surgery group were also identified as DMGs. On opposite, only a few down-regulated (4, 2.4%; Fig. 5A) and up-regulated DEGs (7, 4.1%; Fig. 5B) exclusive for RYGB + SG surgery group were also identified as DMGs. Finally, most of the genes simultaneously identified as DEGs and DMGs common to both surgery groups (126, 16.1%) were down-regulated DEGs, as compared to only 20 (7.1%) up-regulated DEGs.

We further investigated whether differentially methylated CpG sites associated to genes being both DEGs and DMGs were located within critical regions for gene transcription or within intergenic regions. As illustrated in Fig. 5C, an important proportion of differentially hypermethylated CpG sites (37.3%) were located within promoter regions, and an even greater proportion within gene bodies (62.7%). Approximately the same proportions were observed for hypomethylated CpG sites (Fig. 5D). Interestingly, none of the CpG sites was located within intergenic regions, suggesting an actual relationship between differential methylation and gene expression changes in genes identified as both DEGs and DMGs.

### Total weight loss was strongly associated with genes being simultaneously differentially expressed and methylated in BPD-DS

**Fig. 6:**
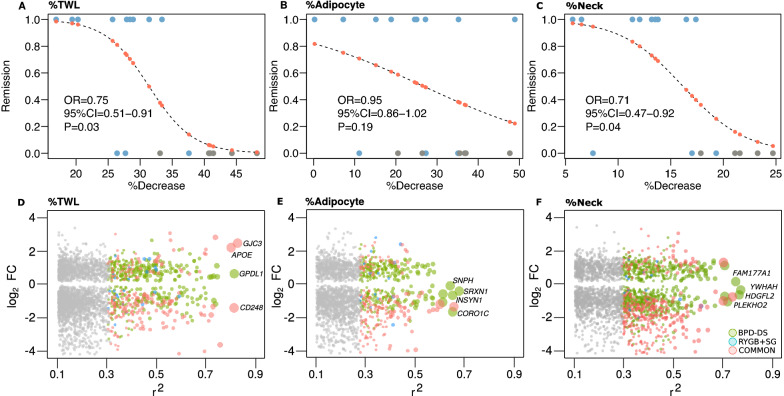
Differentially expressed and methylated genes were associated with weight loss, adipocyte size and neck circumference. **A** to **C** show the predicted probability (red dots from 0 to 1), obtained by binomial logistic regression, of each participant to have a complete (0) or a partial remission (1), based on %TWL, %adipocyte size and %neck circumference. OR is the odds ratio with 95% confidence intervals (CI) and P is the p value for the linear trend of association. Gray and blue dots refer BPD-DS and RYGB + SG, respectively. **D** to F respectively show associations between differentially expressed genes (DEGs) in each surgery group with the percentage of total weight loss (%TWL), adipocyte size change (%Adipocyte) and neck circumference change (%Neck). Green, blue and red dots respectively stand for associations at non-adjusted p < 0.05 with DEGs exclusive to BPD-DS, RYGB + SG or common to both surgery groups. Grey dots represent not significant associations. Dot size is proportional to the magnitude (r^2^) of the association. Results are from multivariate linear regression models adjusted for sex, age and BMI

According to the results showing higher remission rates following BPD-DS, we also found that individuals losing more weight were also those more prone to achieve total remission (OR = 0.75; 95%CI = 0.51–0.91; p = 0.03) (Fig. 6A). Similarly, %TWL was also significantly associated with type 2 diabetes remission (OR = 0.78; 95%CI = 0.62–0.99; p = 0.04) and showed a trend for association with dyslipidemia remission (OR = 0.69; 95%CI = 0.46–1.03; p = 0.07). We then tested whether %TWL was associated with gene expression changes in SAT from baseline to 12 months postoperative. Within DEGs common to both surgeries, a total of 24 up-regulated (8%) and 38 down-regulated DEGs (5%) were significantly associated with %TWL. Among common DEGs, the strongest positive associations were observed for the *GJC3* (r^2^ = 0.84) and *APOE* (r^2^ = 0.82) genes, and a strong inverse correlation was found for *CD248* (r^2^ = 0.83) (Fig. 6D). %TWL was also significantly associated with 75 up-regulated (11%) and 49 down-regulated DEGs (5%) exclusive to BPD-DS, with *GPD1L* showing the strongest positive association (r^2^ = 0.83) (Fig. 6D). None of DEGs exclusive to RYGB + SG was significantly associated with %TWL. Most of down-regulated (33, 67%) and almost half of up-regulated DEGs (36, 48%) exclusive to BPD-DS and showing a significant association with %TWL were also identified as DMGs.

On the other hand, adipocyte size change did not show a significant impact on total remission rates (OR = 0.95; 95%CI = 0.86–1.02; p = 0.19) (Fig. 6B), nor on type 2 diabetes or dyslipidemia remission, and was only significantly associated with four DEGs, all of them being down-regulated DEGs exclusive to BPD-DS: *INSYN1* (r^2^ = 0.68), *SRXN1* (r^2^ = 0.65), *CORO1C* (r^2^ = 0.65) and *SNPH* (r^2^ = 0.64) (Fig. 6B). Interestingly, all of them were also significantly associated with %TWL, and three were also identified as DMGs (*INSYN1*, *CORO1C* and *SNPH*).

Finally, neck circumference change was also found to be a significant predictor of total remission (OR = 0.71; 95%CI = 0.47–0.92; p = 0.04) (Fig. 6C) and dyslipidemia remission (OR = 0.74; 95%CI = 0.55–0.99; p = 0.04), and showed a trend for association with type 2 diabetes remission (OR = 0.80; 95%CI = 0.63–1.01; p = 0.06). Accordingly, 110 up-regulated and 6 down-regulated DEGs exclusive to BPD-DS were significantly associated to this variable, as well as 23 up-regulated and 9 down-regulated DEGs common to both surgery groups, but none were exclusive to RYGB + SG. A total of 47 DEGs significantly associated with neck circumference change were also associated with %TWL.

## Discussion

Following bariatric surgery in any of the procedures analyzed, most of the participants lost 20% or more of their initial weight, which has been suggested as a threshold for intervention success [34, 35]. Also, global transcriptomic and methylomic findings highlighted that, independently of the type of procedure, bariatric surgery induced a profound molecular remodeling in the SAT of patients with severe obesity, mainly through gene down-regulation and hypermethylation. However, weight loss at 12 months postoperative was far more important in participants who underwent BPD-DS, as compared to those undergoing RYGB + SG, which may partly explain the more extensive transcriptomic and methylomic modifications observed in the SAT of BPD-DS participants. It should be noted that less than 35% of all DEGs and only 17% of DMGs were common to both BPD-DS and RYGB + SG surgery groups. Moreover, the extent of gene down-regulation and hypermethylation among common DEGs and DMGs was 50% greater following BPD-DS than after RYGB + SG. Even more striking was that more than 50% of DEGs and almost 80% of DMGs were exclusive to BPD-DS, suggesting more extensive transcriptomic and methylomic modifications in SAT following this surgery, which also represents 70% of genes being simultaneously identified as DEGs and DMGs. Interestingly, an important proportion of differentially methylated CpG sites was located within promoter regions, through which gene expression can be regulated. Part of the methylation profile may be acquired during embryogenesis and is thought to remain stable across the lifespan [36]. However, some methylation marks may also be modified from the exposition to environmental factors such as diet, exercise, obesity, ageing or bariatric surgery [10, 11, 36].

Greater remission rates in type 2 diabetes following BPD-DS in comparison to RYGB and SG procedures have been previously reported [23, 37, 38]. These beneficial effects on type 2 diabetes are maintained over time with slightly more than 90% of the patients who discontinued diabetic therapy 10 years after undergoing BPD-DS surgery [21]. Besides the impressively favorable impact on plasma glucose and insulin levels, other beneficial metabolic shifts have been reported to be more persistent in long-term follow-ups with BPD-DS compared to RYGB, such as improved lipid profile and blood pressure lowering [21, 39, 40]. Whether these greater modifications are due to a more important and sustained weight loss or to more profound metabolic modifications after BPD-DS is still unknown. In this regard, it is worth highlighting that other factors than surgery type itself, such as sex and initial BMI, have been revealed to have a critical impact on weight loss and health outcomes following a bariatric surgery, with heavier men usually having the worst prognosis [41–43]. We also previously observed that men are overrepresented among subjects with a reduced weight loss response after surgery and, more importantly, that initial BMI is the best predictor of weight loss [44]. Concretely, we reported that the probability to show reduced weight loss following bariatric surgery significantly increases with initial BMI and mostly in men. In the present study, we tried to minimize a potential sex bias by keeping similar proportions of men and women among the different procedures, as well as by excluding for the analysis all the transcripts and CpG sites located on sexual chromosomes. Similarly, we used %TWL instead of %EWL as a measure of body weight loss, in an effort to reduce the impact of initial BMI, as recently reported [45]. Also, in order to take into account baseline differences between participants, differential gene expression analysis was performed by using a paired design, and linear as well as logistic models were adjusted for age, sex and initial BMI. Herein, having these considerations into account, we have established a potential link between a greater weight loss reduction with a more extensive transcriptomic and methylomic remodeling in SAT, which ultimately may contribute to these metabolic improvements. Similarly, gene expression changes in SAT were strongly associated with the reduction of neck circumference following bariatric surgery, which was previously suggested to be a reliable predictor of remission rates [46].

In animal models, metabolic changes seem to be mostly attributable to the malabsorptive effect of BPD-DS [47]. Vink et al. [48] have observed that a very-low caloric diet compared to a low caloric diet with similar weight loss induced greater gene expression modifications in pathways related to mitochondrial function, adipogenesis, immunity and inflammation. Thus, it is possible that the more pronounced effects ascertained in this study in BPD-DS compared to RYGB + SG were also partly attributable to a decrease in lipid absorption [49]. Moreover, the jejunum maybe crucial in regulating insulin sensitivity and is completely bypassed in BPD-DS [49]. Among mouse models, a high-fat compared to a low-fat isocaloric diet led to greater modifications in gene expression and methylation [50]. However, mice in the high-fat diet group were also significantly heavier following the diet than mice in the low-fat group [50]. It is not known whether diet or weight gain was responsible to induce these greater changes. In this regard, different bariatric procedures may also lead to distinct changes in diet, but actual data do not allow to test whether these changes are also impacting the present results.

In our study, many significantly enriched biologic processes related to protein translation and ribosomal activity were observed for up-regulated DEGs exclusive to BPD-DS and common to both surgery groups. Enrichments in similar pathways have been previously reported after RYGB [32] and diet-induced weight loss [48]. Genes involved in protein translation are also differently expressed among metabolically unhealthy individuals with obesity [51]. Protein translation may be regulated through an enhanced insulin sensitivity following bariatric surgery or dietary-induced weight loss [52]. For instance, insulin activates eukaryotic initiation and elongation factors, and increases the cellular content of ribosomal proteins [53]*.* Herein, many genes encoding ribosomal proteins (*RPS* and *RPL* genes) were up-regulated. In addition, genes encoding eukaryotic translation initiation factor 4E binding proteins (*EIF4EBP1*, *EIF4EBP2* and *EIF4EBP3* genes) and eukaryotic translation initiation factors (*EIF* genes) were found among the most up-regulated DEGs*.* Although these genes all encode proteins involved in translation, they may also be linked to adiposity, adipogenesis or glucose homeostasis. In fact, some *EIF* genes have been reported to correlate with genes encoding insulin receptor (*INSR*) and insulin receptor substrate-1 (*IRS-1*) [54], which were both also significantly up-regulated in the present study.

More than a simple reservoir of energy surplus, SAT has important endocrine and paracrine functions which regulate many biological processes [55]. Overweight and obesity may lead to SAT dysfunction including several perturbations, as an impaired expandability and adipocyte hypertrophy, altered innate and adaptative immune functions, changes in the secretion of pro- and anti-inflammatory peptides and eventually fibrosis [56, 57]. Transcriptomic modifications in pathways related to immunity and/or inflammation following weight loss, induced either by bariatric surgery or by diet, have been previously observed [27, 28]. Here, almost all the biological processes found to be significantly enriched with down-regulated DEGs were related to immune-related functions. Interestingly, most of such pathways were also significantly enriched with hypermethylated DMGs, pointing to a marked rearrangement of inflammation and immune molecular processes in the SAT of study participants. Although this effect was observed independently of the bariatric procedure analyzed, it was particularly pronounced following BPD-DS. By contrast, the fact that none of these pathways was significantly enriched with DEGs or DMGs exclusive to RYGB + SG emphasizes the procedure-specific nature of many of these molecular changes. Among significantly enriched immune-related pathways, biological processes such as T cell activation, leukocyte cell–cell adhesion, neutrophil activation and ERK1/2 cascades were significantly enriched. It has been observed that individuals with obesity have an increased quantity of T lymphocytes in their SAT [58]. Moreover, T regulatory lymphocytes have been shown to be reduced following bariatric surgery [59]. Neutrophils are an essential component of the innate immune response. Following RYGB, Poitou et al. [60] identified several DEGs which were related to neutrophil-mediated inflammation. In their study [60], DEGs related to neutrophils function or activity included *S100A8*, *S100A9*, *S100A12*, *CD300E*, *VNN2*, *FPR2* and *APOBEC3A*, which were all also differentially expressed in both surgery groups of the present study, but around twice as much in BPD-DS than in RYGB + SG. Interestingly, Kerr et al. [32] have observed that there were continuously down-regulated genes 5 years following RYGB, despite significant weight regain occurring from 2 to 5 years postoperative. The authors observed that these continuously down-regulated genes were involved in biological processes such as cytokine production, cell chemotaxis and neutrophil activation [32], suggesting that these gene modifications might not be linked exclusively to weight variations. Moreover, these long-term changes in gene expression may be sustained through epigenetic mechanisms.

Two genes encoding for cytokines were among DEGs with the greatest change extent for both surgery groups, *CSF3*, which encodes for colony stimulating factor 3, a cytokine that has been reported to be elevated among individuals with obesity [61], and *IL6*, which is a well-known cytokine involved in inflammation. Following weight loss induced either by diet or bariatric surgery, a down-regulation in gene expression was previously noticed for genes such as *CCL2* [28], *NFKB1* [32], *NLRP3* [32], *HIF1A* [27], *CLEC7A* [27] and *IL4R* [27]. These genes have also been found to be significantly down-regulated herein. Among them, *HIF1A* may indirectly activate *NLRP3*, which encodes for NLRP3 inflammasomes, contributing to the inflammatory responses via IL-1β activation, which is down-regulated to a greater extent in BPD-DS in this study, and could be of key importance in the development of type 2 diabetes [62].

Biological processes significantly enriched with hypomethylated DMGs were mostly related to extracellular structure and matrix organization, actin filament organization, cell-substrate and matrix adhesion, as well as cell-substrate junction and assembly, among others. These changes were again more pronounced following BPD-DS. Kelehmainen et al.[63] reported a down-regulation of DEGs involved in extracellular matrix following weight loss. The excessive accumulation of extracellular matrix components associated with obesity can lead to adipose tissue fibrosis which contributes to the dysfunction of adipocytes [64]. Moreover, higher SAT fibrosis may lessen the weight loss response following RYGB [64]. In the present study, these pathways were hypomethylated but not up-regulated. Thus, the functional impact of this hypomethylation remains unknown. It is possible that changes in gene expression were transient and no longer present at 12 months postoperative, since they potentially occurred earlier following the bariatric surgery, as previously shown in skeletal muscle [31].

Among significantly down-regulated DEGs common to both surgery groups, a strong inverse association with %TWL was observed for *CD248*, a gene which encodes for tumor endothelial marker 1/endosialin, a transmembrane glycoprotein known to be expressed in proliferating tissues, especially during embryogenesis, tumor growth and inflammatory lesions [65]. More recently, Petrus et al. [66] have demonstrated that *CD248* is up-regulated in the SAT of individuals with obesity and insulin resistance and is potentially involved in the response to hypoxia. They reported that both *CCL2* and *IL6*, respectively involved in extracellular matrix remodeling and inflammation, were correlated positively with *CD248* gene expression [66]. Among up-regulated DEGs common to both surgery groups, *GJC3* was associated with %TWL and it has been reported to be down-regulated in obesity [67]. On the other hand, *GPD1L* was the up-regulated DEG exclusive to BPD-DS most strongly associated with %TWL. In a long-term follow-up study, *GPD1L* was reported to be regulated by weight loss and regain after RYGB [32]. Furthermore, *GPD1L* was recently identified as potentially playing a causal role in obesity and insulin resistance [68]. During weight loss and weight maintenance induced by a low caloric diet, *GPD1L* was found to be up-regulated, while being down-regulated during weight gain induced by a high-fat diet [68]. It is worth noting that in the present study, most of DEGs exclusive to BPD-DS and associated with %TWL were also identified as DMGs, while none of the DEGs exclusive to RYGB + SG group were significantly associated to %TWL. Moreover, a total of four down-regulated DEGs exclusive to BPD-DS also showed an association with adipocyte size change. Among them, *CORO1C*, a gene recently identified to be up-regulated in the SAT of individuals with obesity [69], was found to be closely linked to %TWL and was also identified as hypomethylated. From a broader perspective, methylomic changes observed in this study were mostly exclusive to BPD-DS, which points to an epigenetic-mediated mechanism by which gene expression changes in SAT may occur in a greater extent in patients undergoing this type of surgery.

Present findings thus provide evidence that BPD-DS induce larger methylomic and transcriptomic modifications than RYGB + SG, which may be partly explained by greater weight loss and malabsorption created by this surgical approach. However, it is also possible that BPD-DS participants, who had higher BMI and waist circumference before surgery, started with a more deteriorated metabolic profile than participants who underwent RYGB or SG, which ultimately led to the more extensive transcriptomic and methylomic modifications observed. Results shown herein were obtained at 12 months postoperative, and it is possible that participants may still be losing weight or not being weight stable, which could affect transcriptome and methylome profiles. However, it has been reported that weight loss is as its nadir around 12 to 18 months following either RYGB [70] or BPD-DS [71].

## Conclusions

To our knowledge, this is the first study examining the impact of bariatric surgery on SAT transcriptomic and methylomic profiles by using two high throughput technologies, RNA sequencing and genome-wide DNA methylation analysis. Our findings provide a novel overview of the transcriptomic and methylomic changes taking place 12 months following a bariatric surgery, concretely for BPD-DS compared to RYGB and SG. These results also confirm those obtained in previous transcriptomic studies following RYGB and SG. For instance, many of the enriched biological pathways found herein are shared with those previously found but have been observed to be of greater magnitude following BPD-DS. Globally, enriched biological processes in SAT following BPD-DS pointed to a strong decrease in immune and inflammatory responses and to an increase in protein translation, as well as to a shift towards modifications in other components of SAT, such as extracellular structure and actin filaments. These results will contribute to a better understanding of the metabolic pathways involved in the response to bariatric surgery and will eventually lead to the development of potential gene targets for the treatment of obesity and its related complications.

## Methods

### Study population

A total of 32 subjects with severe obesity, defined as BMI greater than or equal to 35 kg/m^2^, and aged between 18 and 60 years old, were recruited from the bariatric surgery clinic of the *Institut universitaire de cardiologie et de pneumologie de Québec* (IUCPQ). Recruitment occurred from September 2015 to November 2017. Exclusion criteria included pregnancy or desired pregnancy during the study; previous esophageal, digestive or bariatric surgery; abnormal bowel habits including irritable bowel syndrome, unexplained intermittent vomiting, severe abdominal pain, as well as chronic diarrhea or constipation in the last 60 days; history of gastric or duodenal ulcers; hypoalbuminemia (< 35 g/L); history of renal, hepatic, cardiac or pulmonary severe disease; evidence of psychiatric problems that may affect the capacity to understand the project and comply with the medical, surgical and/or behavioral recommendations; history of drug use or alcohol abuse in the last 12 months and during the study, as well as history of gastrointestinal inflammatory diseases. Of the initial 32 participants enrolled in the study, analyses were finally conducted on the 21 subjects for which SAT biopsies were successfully performed during bariatric surgery and 12 months later, including 10 men and 11 women. The clinical trial REMISSION is registered at Clinicaltrials.gov (NCT02390973).

### Short-term prospective study protocol

Clinical exams including fasting biochemistry and anthropometric measurements were performed preoperatively and 12 months following the bariatric surgery. Participants underwent either BPD-DS, RYGB or SG according to National Institutes of Health (NIH) consensus for gastrointestinal surgery criteria [72] and based on the surgeon–patient’s choice at the bariatric surgery clinic of IUCPQ. After the surgery, participants followed a standardized postoperative protocol including feeding and a supplementation with vitamins and minerals. A detailed description of the BDP-DS procedure is given elsewhere [73].

### Anthropometric measurements

Height and body weight were measured preoperatively and 12 months following bariatric surgery, and BMI was calculated as the weight in kilograms divided by the height in square meters. As recommended by the American Society for Metabolic and Bariatric Surgery (ASMBS) for reporting weight loss outcomes [8], we present the following information in the results section: mean initial BMI, change in BMI, %TWL and %EWL. %TWL was calculated as follows: [(Initial Weight)—(Postoperative Weight)] / [(Initial Weight)] * 100. %EWL was calculated as follows: [(Initial Weight)—(Postoperative Weight)] / [(Initial Weight)—(Ideal Weight)] * 100. Ideal weight is defined as the weight corresponding to a BMI of 23 kg/m^2^. A recent systematic review[75]investigated weight loss outcomes of RYGB and SG concluded that %TWL should be preferred over %EWL to minimize baseline BMI influence [45]. In this view, %TWL was used in the present study as the main weight loss outcome. Neck circumference, recently reported as a reliable predictor for the success of bariatric surgery [46], was also measured preoperatively and 12 months following the surgery.

### Remission measurements

Overnight fasting blood samples were collected in the morning of each visit. Briefly, cholesterol and triglyceride levels were measured in plasma and lipoprotein fractions with a Technicon RA analyzer (Bayer, Etobicoke, ON, Canada) using enzymatic methods. Dyslipidemia remission was qualified according to plasma levels of low- (LDL) and high-density lipoproteins (HDL), total cholesterol and triglycerides [8]. Glucose was measured using the glucose oxidase method and insulin was quantified by radioimmunoassay (Linco Research, St. Charles, MO, US). The homeostasis model assessment of insulin resistance (HOMA-IR) index was calculated using the following formula: fasting insulin (μU/mL) * fasting glucose (mmol/L)/22.5. Diabetes remission was defined as suggested by the ASMBS [8] (HbA1c < 6.0% and fasting glycemia < 7.0 mmol/L in the absence of anti-diabetic pharmacological treatment), partial remission (HbA1c 6%-6.4% and fasting glycemia 5.6 mmol/L-6.9 mmol/L), improvement (statistical reduction in HbA1c and fasting glycemia not meeting criteria for remission or decrease in antidiabetic medications requirement). For comparative purposes, intermediate dyslipidemia and type 2 diabetes remission rates were grouped into two larger groups: partial and complete remission. A novel parameter called total remission was defined herein as the complete remission of both type 2 diabetes and dyslipidemia.

### Adipose tissue sampling

Samples of SAT were collected at the site of the surgical incision in the lower abdomen. Immediately following surgical removal, fresh adipose tissue samples were carried to the laboratory where a portion of each sample was flash frozen in liquid nitrogen and stored at − 80 °C for further RNA and DNA extraction. Another portion of SAT sample was digested and used for adipocyte isolation and cell sizing. Briefly, tissue samples were digested with collagenase in Krebs–Ringer-Henseleit buffer for 45 min at 37 °C according to a modified version of the Rodbell method, as previously described [74]. Cell suspensions were filtered through nylon mesh and washed with Krebs–Ringer-Henseleit buffer. To determine adipocyte diameter, pictures of 250 cells were taken with a light microscope and analyzed with Scion Image software [74].

### RNA sequencing

Total RNA from SAT biopsies obtained before and 12 months following the surgery was extracted using a RNeasy Lipid Tissue Mini Kit (Qiagen, Mississauga, ON, Canada) following the manufacturer’s instructions and treated with DNase (Qiagen) to avoid DNA contamination. RNA integrity was evaluated using the Agilent 2100 Bioanalyzer system (Agilent, Santa Clara, CA, US). RNA sequencing was performed at the McGill University and Génome Québec Innovation Centre (MUGQIC). Library preparation was carried out using the Illumina NEB stranded mRNA library preparation kit (Illumina, San Diego, CA, US) and sequencing was performed on the Illumina NovaSeq 6000 S4 platform (Illumina) using 100 bp paired-end reads. Raw reads were first trimmed at 50 bases and at a Phred quality score of 30 using Trim Galore! (v0.6.4) [75], a wrapper tool around Cutadapt (v3.2) [76] and FastQC (v0.11.9) [77]⁠. Read quantification was performed using kallisto (v0.46.2) [78] with 100 bootstraps. Reads were aligned to the GRCh38 human reference transcriptome and transcripts located on sexual chromosomes were excluded for further analyses. The obtained transcript counts were used to infer gene-level abundance estimates with the tximport (v1.20.0) R package [79]. Gene expression was then normalized, and lowly expressed genes were filtered out with the filterByExpr function in edgeR (v3.34.0) [80], leaving a total of 18 862 genes for further analyses. Differential gene expression analysis was performed between pre-surgical and 12-month follow-up levels in edgeR using a paired design, which can be viewed as a generalization of a paired t-test. DEGs were considered at false discovery rate (FDR)-corrected p-value < 0.05 and fold change (FC) > 1.5.

### Genome-wide methylation analysis

Genomic DNA of the 21 study participants was extracted from 200 mg of SAT biopsy samples obtained before and 12 months following surgery using the DNeasy Blood & Tissue kit (Qiagen). Following quantification of DNA using both NanoDrop Spectrophotometer (Thermo Scientific, Wilmington, DE, US) and PicoGreen DNA methods, DNA (1 μg) was bisulfite converted. Quantitative genome-wide methylation analysis was conducted using the EPIC platform (Illumina), interrogating over 850 000 CpG sites at single-nucleotide resolution. Methylation arrays were processed at the MUGQIC according to manufacturer’s instructions. All probes with low detection p-values (< 0.05) were removed, as well as those located in sex chromosomes. Polymorphic and cross-reactive probes were also excluded, leaving a total of 774 177 probes for further differential methylation analyses. Methylation data was normalized with the quantile method, as previously described [81], using the minfi (v1.38.0) R package [82]⁠. Methylation levels (beta values, β) were estimated as the ratio of signal intensity of the methylated alleles to the sum of methylated and unmethylated intensity signals. The β values varied from 0 (no methylation) to 1 (100% methylation). Differentially methylated CpG sites were considered at FDR-corrected p-value < 0.05 and FC > 1.5. (DMGs were defined as loci with at least one differentially methylated mapped CpG site.

### Pathway enrichment analysis

The functional significance of DEGs and DMGs was explored by pathway enrichment analysis using the clusterProfiler v3.16.0 R package [83]. The clusterProfiler package implements statistical methods to analyze functional profiles of genes and gene clusters and produces FDR-adjusted p-values to show significantly enriched pathways. The Gene Ontology Biological Processes (GO-BP) database was used for functional enrichment analysis. Pathway enrichment analysis was performed with DEGs and DMGs common to both surgery groups analyzed, as well as with those exclusive for BPD-DS and RYGB + SG. Pathways were considered significantly enriched at FDR-adjusted p-value < 0.05 and composed with at least 20 DEGs or DMGs.

### Statistics

Clinical data were checked for normality with the Kolmogorov–Smirnov test and two-group comparisons were tested with two-tailed Student’s t test for independent samples. Fisher exact test was used to compare remission success rates between surgery groups, as well as the proportion of men and women. Multivariate linear models adjusted by sex, age and BMI were implemented to test for associations between gene expression and gene methylation levels with %TWL, adipocyte size change and neck circumference change. Linear associations were considered significant when FDR-corrected p-value < 0.05. Binomial logistic regression was used to predict the probability of total remission success set as a dichotomous variable. %TWL, adipocyte size change and neck circumference change were tested as independent predictors, with age, sex and BMI set as covariables. All the statistical analysis were implemented in R (v4.1.0) [84]⁠.

## Supplementary Information

**Additional file 1: Table S1.** Participants characteristics. **Figure S1.** Subcutaneous adipose tissue morphology before and after the bariatric surgery.

## Data Availability

The datasets generated during the current study are not publicly available due to privacy and confidentiality reasons but are available from the corresponding author on reasonable request.
