# Supplementary material for: Molecular remodeling of adipose tissue is associated with metabolic recovery after weight loss surgery
Source: J Transl Med. 2022 Jun 23;20:283. doi: 10.1186/s12967-022-03485-6 (PMC9219157; doi:10.1186/s12967-022-03485-6)
Supplement: Supplementary file 1 — Additional file 1: Table S1. Participants characteristics. Figure S1. Subcutaneous adipose tissue morphology before and after the bariatric surgery. [file 12967_2022_3485_MOESM1_ESM.pdf]

Molecular remodeling of adipose tissue is associated with metabolic recovery after weight loss surgery

Annie Bouchard-Mercier[1,2†] (annie.bouchard@valbiotis.com)
Juan de Toro-Martín[1,2†] (juan.de-toro-martin.1@ulaval.ca)
Mélanie Nadeau[3] (melanie.nadeau@criucpq.ulaval.ca)
Odette Lescelleur[3] (odette.lescelleur@criucpq.ulaval.ca)
Stéfane Lebel[3] (stefane.lebel@fmed.ulaval.ca)
Denis Richard[3] (denis.richard@criucpq.ulaval.ca)
Laurent Biertho[3] (laurent.biertho@criucpq.ulaval.ca)
André Tchernof[3] (andre.tchernof@criucpq.ulaval.ca)
Marie-Claude Vohl[1,2*] (marie-claude.vohl@fsaa.ulaval.ca)

[1]School of Nutrition, Université Laval, 2440 Hochelaga Blvd, Quebec City, Quebec, Canada, G1V 0A6.
[2]Centre Nutrition, Santé et Société (NUTRISS)-Institut sur la nutrition et les aliments fonctionnels (INAF), Université Laval, 2440 Hochelaga Blvd, Quebec City, Quebec, Canada, G1V 0A6.
[3]Centre de recherche de l'Institut universitaire de cardiologie et de pneumologie de Québec (IUCPQ), 2725 chemin Sainte-Foy, Quebec City, Quebec, Canada, G1V 4G5.

†These authors share first authorship. The first author was determined alphabetically.

*Corresponding author:
Marie-Claude Vohl
Institut sur la nutrition et les aliments fonctionnels (INAF)
Université Laval – Pavillon des Services (suite 2729K)
G1V 0A6
Quebec (QC) – Canada
418 656-2131 (ext 4676)
marie-claude.vohl@fsaa.ulaval.ca

Supplementary Material

Table S1. Participants characteristics

	Preoperative							Postoperative						
	**SG**	**RYGB**	**BPD-DS**	**P-value**	**SG vs RYGB**	**SG vs BPD-DS**	**RYGB vs BPD-DS**	**SG**	**RYGB**	**BPD-DS**	**P-value**	**SG vs RYGB**	**SG vs BPD-DS**	**RYGB vs BPD-DS**
N (male)	9 (5)	5 (3)	7 (2)	0.5										
Age (years)	53.4 ± 8.1	55.7 ± 7.8	47.8 ± 7.1	0.4	0.9	0.4	0.2							
Height (cm)	168.6 ± 8.6	166.4 ± 9.7	167.1 ± 8.9	0.7	0.9	0.9	1.0							
Body weight (kg)	123.5 ± 18	106 ± 9.1	139.8 ± 15.3	**0.01**	0.1	0.1	**0.004**	90.8 ± 12.8	71.5 ± 10.1	81.4 ± 10.0	**0.02**	**0.02**	0.3	0.3
BMI (kg/m^2)	43.3 ± 3.8	38.4 ± 2.8	50.2 ± 5.5	**0.005**	0.1	**0.01**	**0.0004**	31.9 ± 3.5	25.8 ± 2.5	29.3 ± 4.1	**0.02**	**0.02**	0.3	0.2
ΔBMI								-11.4 ± 3.1	-12.6 ± 2.6	-20.9 ± -2.9	**0.0001**	0.7	**0.00001**	**0.0004**
%TWL								26.2 ± 6.5	31.9 ± 6.4	41.7 ± 4.6	**0.0003**	0.2	**0.0002**	**0.03**
%EWL								56.7 ± 14.8	80.6 ± 16.1	78.6 ± 15.1	**0.008**	**0.03**	**0.03**	1.0
Neck circ. (cm)	44.7 ± 2	43.6 ± 3.8	45.1 ± 3.3	0.4	0.8	1.0	0.7	39.7 ± 2.6	36.8 ± 2.6	35.2 ± 2.8	**0.01**	0.2	**0.01**	0.6
Waist circ. (cm)	138.7 ± 9.3	125.9 ± 5.2	147.9 ± 9.8	**0.002**	**0.04**	0.1	**0.001**	111.7 ± 8.6	93.4 ± 5.5	104.1 ± 8.5	**0.006**	**0.002**	0.2	0.1
Fat mass (kg)	59.4 ± 13.2	47.9 ± 7.8	78.3 ± 7.5	**0.003**	0.1	**0.006**	**0.0003**	30.6 ± 11.2	17.2 ± 5.1	26.8 ± 9.1	0.1	0.1	0.7	0.2
Fat free mass (kg)	65.6 ± 10.8	60.8 ± 11.7	66.1 ± 6.2	0.2	0.7	1.0	0.6	58.5 ± 10.7	54.7 ± 12.2	56.3 ± 5.1	0.4	0.8	0.9	1.0
Adipocyte size (μm)	86.1 ± 7.4	84.4 ± 10.8	88.8 ± 5.1	0.5	0.9	0.8	0.6	70.1 ± 5.3	55.3 ± 5.1	58.5 ± 7.9	**0.0002**	**0.001**	**0.006**	0.7
SBP	136 ± 13.2	139.8 ± 26.6	143.1 ± 16.5	0.8	0.9	0.7	0.9	128.9 ± 14.2	142.2 ± 29.2	129.9 ± 12.3	0.5	0.4	1.0	0.5
DBP	81.9 ± 4.5	81.6 ± 10.7	82.7 ± 6.8	1.0	1.0	1.0	1.0	76.6 ± 11.8	82.2 ± 16.9	74.3 ± 8.8	0.4	0.7	0.9	0.5

SG, sleeve gastrectomy. RYGB, Roux-en-Y gastric bypass. BPD-DS, biliopancreatic diversion with duodenal switch. N, number of participants. BMI, body mass index. ΔBMI, delta BMI, %TWL, percentage of total body weight loss, %EWL, percentage of excess body weight loss. Circ, circumference. SBP and DBP, systolic and diastolic blood pressure, respectively. P-values are obtained from one-way ANOVA adjusted by age, sex and BMI. Post-hoc Tukey test was used for multiple comparisons among surgery group

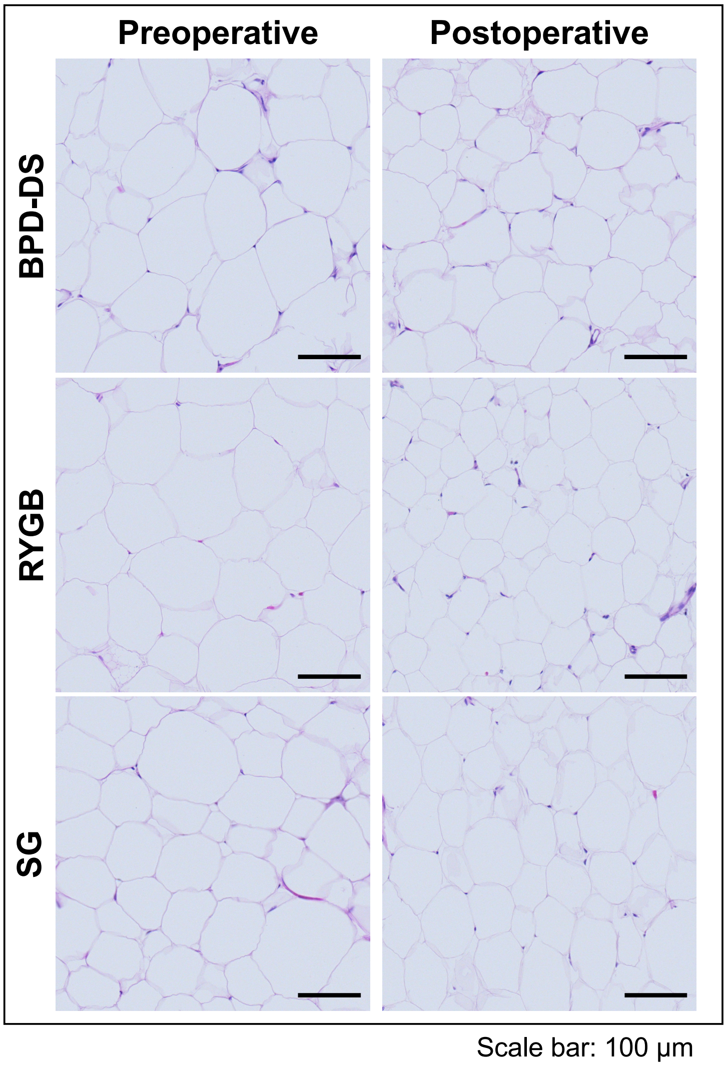

Figure S1. Subcutaneous adipose tissue morphology before and after the bariatric surgery. Micrographs were taken from histological sections of subcutaneous adipose tissue biopsies obtained during surgery (preoperative) and 12 months after (postoperative) and stained with hematoxylin/eosin. Preoperative and postoperative images shown were taken from the same participant. All the representative images shown were taken from women. The mean adipocyte sizes ± standard deviation of each participant are given below. From left to right and top to bottom: biliopancreatic diversion with duodenal switch (BPD-DS, 88.3 ± 22.6 vs 55.9 ± 15.9), Roux-en-Y gastric bypass (RYGB, 84.9 ± 19.8 vs 59.5 ± 13.9) and sleeve gastrectomy (SG, 82.4 ± 20.7 vs 69.9 ± 13.8). All images were taken with a light microscope at 20x magnification.
